# Higher mortality of patients on haemodialysis with pancreatic diabetes compared to type 2-diabetes

**DOI:** 10.1186/1758-5996-4-9

**Published:** 2012-03-23

**Authors:** Gert Bodlaj, Georg Biesenbach

**Affiliations:** 1 From the 2nd Department of Medicine, Section Nephrology, General Hospital Linz, Austria

**Keywords:** Pancreatic diabetes, End-stage renal disease, Outcome

## Abstract

In rare cases (1-8%) diabetic patients with end-stage renal disease (ESRD) suffer from diabetic nephropathy (dNP) due to pancreatic diabetes mellitus (PDM). Aim of this study was to investigate differences in the outcome of patients with PDM and those with type 2 diabetes.

In a retrospective study we evaluated 96 diabetic patients, who started hemodialysis (HD) in our dialysis centre (1997-2005). In 12 patients PMD was diagnosed, and 84 patients had type 2 diabetes. In both groups we compared vascular risk factors and prevalence of vascular diseases at the start of dialysis. We also evaluated incidence of malnutrition, and 5-year survival in both patient groups.

The vascular risk factors were similar in both patient groups, also the prevalence of vascular diseases at the initiation of HD was similar in both groups. In the patients with PDM the mean BMI (kg/m^2^) was lower (22 + 3 versus 25 + 3), and also their serum albumin was lower (2.7 + 0.3 versus 3.4 + 0.3 g/dl, p < 0.05). Four of these patients (33%) developed malnutrition (BMI < 18.5). In the patients with PDM the age adjusted 5-year survival was significantly lower (8% versus 27%, p < 0.05) than in the type 2 diabetic patients.

Conclusions in HD-treated patients with type 2 diabetes or PDM the prevalence of vascular diseases was not significantly different. The lower survival of PDM patients can be related to poor nutrition status.

## Introduction

In rare cases (1-8%) patients w/ith pancreatic diabetes (PDM) develop diabetic nephropathy (dNP) with ESRD [1-4] The aim of this retrospective study was to investigate differences in the clinical outcome of uremic patients with dNP due to type 2 diabetes or PDM. There are only few data in the literature dealing with PDM and ESRD [3]. In a recent study was reported, that only 1-5% of diabetic patients with ESRD suffer from pancreatic diabetes [Choudhuri et al. 2009]. Aim of our study was to investigate prevalence of atherosclerosis and to evaluate differences in the outcome of patients with PDM and those with type 2 diabetes.

## Patients

During the years 1997-2005 we selected 144 type 2 diabetic patients who started chronic hemodialysis (HD) in our dialysis centre. We excluded patients with death within the first three months of HD (n = 24), kidney transplantation (n = 8) and patients (n = 16) with vascular nephropathy (vNP). Finally, a total of 96 patients with dNP were included in a retrospective study, 12 patients of them with dNP due to PDM versus 84 patients with dNP caused by type 2 diabetes. The diagnosis of PDM was based on hyperglycemia after recurrent onset of acute pancreatitis and/or pancreas resection. The diagnosis of dNP was based on proteinuria, normal urine sediment, normal kidney size and long acting diabetes. The diagnosis of vNP was defined as renal atherosclerosis and/or shrinkage of a kidney.

In both groups we compared major vascular risk factors and prevalence of vascular diseases at the start of dialysis. We also evaluated incidence of malnutrition, and 5-year survival in both patient groups as well as the prevalence of vascular diseases at the start of HD. Diagnosis of cerebrovascular disease (CVD), coronary artery disease (CAD) and of peripheral vascular disease (PVD) was described in the literature [5]. We also investigated the nutritional status, the frequency of malnutrition and of exocrine pancreatic insufficiency, associated with chronic diarrhoea [6]. The observation period of the study was five years. Thus, in our analysis, the endpoint of the study was death by any cause, while the basal disease PDM or type 2 diabetes were considered as covariables.

For statistical analysis the SPSS for Windows statistical program was used. The statistical methods included the chi-squared test for comparing differences between the groups, and the unpaired student's test for testing unequal variances as well as the Kuscal test for comparisons among the groups. A p-value of less than 0.05 was considered significant.

## Results

At the time of initiating HD treatment the patients with PDM were significantly younger than those with type 2 diabetes (mean age 56 versus 63 years). The vascular risk factors were similar in both groups, only cholesterol was lower in the PDM patients; the HbA1c values were not different (mean control levels 7.5 versus 7.6%), however, the patients with PDM required significantly less insulin. All baseline data of both patient groups are presented in Table 1. In our patients it can be assumed, that dNP was the cause of ESRD. The clinical diagnosis of dNP was confirmed by histological investigations in 44 diabetic patients.

**Table 1:** Baseline data at the start of dialysis

	Pancreatic diabetes	Type 2 diabetes
	(n = 12)	(n = 84)
Age (years)	56 ± 6*	63 ± 8*
Sex (female %)	50%	52%
Diabetes duration (years)	17 ± 7	18 ± 6
HbA1c (%)	7.5 ± 1.3	7.6 ± 1.1
Body weight (kg)	59 ± 7**	70 ± 11**
BMI (kg/m^2^)	22 ± 3*	25 ± 4*
C-peptide basal (ng/ml)	0.9 ± 0.3*	1.9 ± 0.6*
Systolic blood pressure (mm Hg)	136 ± 12	137 ± 10
Diastolic blood pressure (mm Hg)	86 ± 9	87 ± 8
Antidiabetic therapy (%)		
Diet and sulfonylurea	0	5%
Conventional insulin therapy	50%	44%
Intensified insulin therapy	50%	41%
Insulin dose (IU/day)	22+11*	32+11*
Vascular access (n/%)		
Arteriovenous fistula	10 (83%)	64 (76%)
Permcath-dialysis catheter	2 (17/%)	20 (24%)
Insulin dose (IU/day)	22 ± 11*	32 ± 16*
Duration of dialysis (hours//week)	13 (12-15)	13 (12-15)
Kt/V (three month after start of HD)	1.3	1.4

p < 0.05, **p < 0.01. Data are expressed as x+SD, x (range) or %

The prevalence of macrovascular diseases at the start of dialysis was not significantly different. The prevalence of CAD was 66% in the type 2 diabetic patients versus 50% in the group. with PDM. The prevalence of CVD was 33 versus 45% and PVD 50 versus 45%.

The nutritional status at the start of HD was poor in the PDM patients. The frequency of underweight (BMI < 21) was 50% versus 9% (p < 0.05). Four patients with PDM (33%) developed malnutrition (BMI < 18.5). In six patients an exocrine pancreatic insufficiency could be observed (50%). The prognostic parameter s-albumin was significantly lower in the PDM group (2.7 + 0.3 versus 3.4 + 0.3 g/l, p < 0.05). The 5- year survival was significantly lower in the group with PDM 8% versus 27% (p < 0.05) The observation period of the study was five years. We measured the 5-year - *absolute *survival rates, which describe the percentage of patients that are alive five years after their disease is diagnosed. The most common causes of death were cardiovascular events, in the PDM group 60% versus 65% in the type 2 diabetic patients. Infection was cause of death in 27% of the PDM patients and 21% of the patients with type 2 diabetes (NS).

## Discussion

Diabetes caused by exocrine pancreatic disease appears to be underestimated and may comprise 8% or more of the general diabetic patient population [4] In our study the causes of PDM were chronic fibrocalculous pancreatitis (n = 8) and pancreas resection due to pancreas neoplasma (n = 4) The vascular risk factors were similar in both patient groups. The traditional risk factors and the non-traditional risk factors [7] are shown in Table 2. Parathormon (PTH) was no routine measurement in earlier years. PTH values were available in 68% of all patients. ESRD patients with PDM versus type 2 diabetes show a similar vascular risk profile and a comparable high prevalence of vascular diseases, though patients with PDM were younger. The CRP values, considered as vascular risk factors, were slightly higher in the patients with PDM, but difference was not significant. Similar data are described in the literature [8]. All prognostic parameters are also summarized in Table 2. It can be assumed that the low patient survival is associated with the poor nutrition status of the PDM patients. This study has several limitations particularly ther small number of PDM patients, the absence of data on PTH-levels and residual renal function, and the absence of more accurate methods of nutritional assesment However, our results suggest that malnutrition is possible explanation to the outcome differences.

**Table 2:** Traditional and non-traditional risk factors and prescribed medication

	Pancreatic diabetes	Type 2 diabetes
Traditional vascular risk factors		
Fasting blood glucose (mg/dl)	146 ± 22	14 ± 22
Systolic blood pressure (mm Hg)	134 ± 12	136 ± 12
Diastolic blood pressure (mm Hg)	82 ± 7	84 ± 8
Uncontrolled hypertension	83%	90%
Cholesterol (mg/dl)	142 ± 42**	196 ± 36**
Smoking	33	30
Non-traditional risk factors		
BMI < 21 (kg/m^2^)	50%	9%
BMI < 18.5 (malnutrition)	3 3%	0
CRP (ng/ml)	1.4 ± 0.9	0.8 ± 0.6
Serum albumin (g/dl)	2.7 ± 0.3*	3.4+0.3*
Hemoglobin (gm/dl)	10 (8-12)	11 (8-13)
Triglycerides (mg/dl)	123 ± 44	156 ± 42
Fluid overload (ultrafiltration necessary)	33%	24%
Prescribed medication		
Iron substitution	27%	17%
25-hydroxy vitamin D	66%	43%
Statin	33%	44%
Aspirin	33%	29%
ACE inhibitors	50%	52%

*p < 0.05, **p < 0.01 Data are expressed as x+SD, x (range) or %

## Conclusion

The prevalence of atherosclerosis is not significantly different in both, patients on hemodialysis with PDM versus type2 diabetes. In patients with PDM the 5-year surviv l is lower than in the type 2 diabetic patients. We confirm, that there are limitations of our study, due to the retrospective analysis of the data and due to the small patient group with PDM.

## Competing interests

The authors declare that they have no competing interests.

## Authors' contributions

GB drafted the manuscript. GB revised the manusript. Both authors read and approved the final manuscript.
